# Supplementary material for: PPM1D mutations silence NAPRT gene expression and confer NAMPT inhibitor sensitivity in glioma
Source: Nat Commun. 2019 Aug 22;10:3790. doi: 10.1038/s41467-019-11732-6 (PMC6706443; doi:10.1038/s41467-019-11732-6)
Supplement: Supplementary file 1 — Supplementary Information [file 41467_2019_11732_MOESM1_ESM.pdf]

# Supplementary Information

# For

# PPM1D Mutations Silence NAPRT Gene Expression and Confer Exquisite NAMPT Inhibitor Sensitivity in Glioma

**Authors**: Nathan R. Fons[1,2], Ranjini K. Sundaram[2], Gregory A. Breuer[1,2], Sen Peng[3], Ryan L. McLean[2], Aravind N. Kalathil[2], Mark Schmidt[4], Diana M. Carvalho[5], Alan Mackay[5], Chris Jones[5], Ángel M. Carcaboso[6], Javad Nazarian[7], Michael E. Berens[3*], Charles Brenner[4*], Ranjit S. Bindra[1,2*]

**Supplementary Fig. 1.** *PPM1D* mutant astrocytes are exquisitely sensitive to NAMPT inhibitors.

**Supplementary Fig. 2.** NAD metabolome depression in PPM1D$^{trnc.}$ astrocytes results in NAMPT inhibitor sensitivity

**Supplementary Fig. 3.** NAPRT deficiency drives sensitivity of *PPM1D* mutant astrocytes to NAMPT inhibitors.

**Supplementary Fig. 4.** Patient-derived SU-DIPG-XXXV neurosphere cell line possesses a truncating *PPM1D* mutation and is sensitive to NAMPT inhibitors.

**Supplementary Fig. 5.** U2OS and MCF7 cell lines contain *PPM1D* alterations, silence *NAPRT* transcription, and are sensitive to NAMPT inhibition.

**Supplementary Fig. 6.** Model DIPG lines with *PPM1D* mutations have reduced NAPRT expression and maintain p53 expression.

**Supplementary Fig. 7.** Mutant PPM1D-induced hypermethylation is distinct from G-CIMP found in *IDH1* mutant astrocytes.

**Supplementary Fig. 8.** *In vivo* efficacy of NAMPT inhibitors in *PPM1D* mutant tumors.

**Supplementary Fig. 9.** Applicability of NAMPT inhibitors for the treatment of *PPM1D* mutant, non-glioma tumors.

**Supplementary Table 1.** Synthetic lethal drug screen compounds and $IC_{50}$ ratios.

**Supplementary Table 2.** Oligos and siRNA target sequences.

**Supplementary Figure 1.**

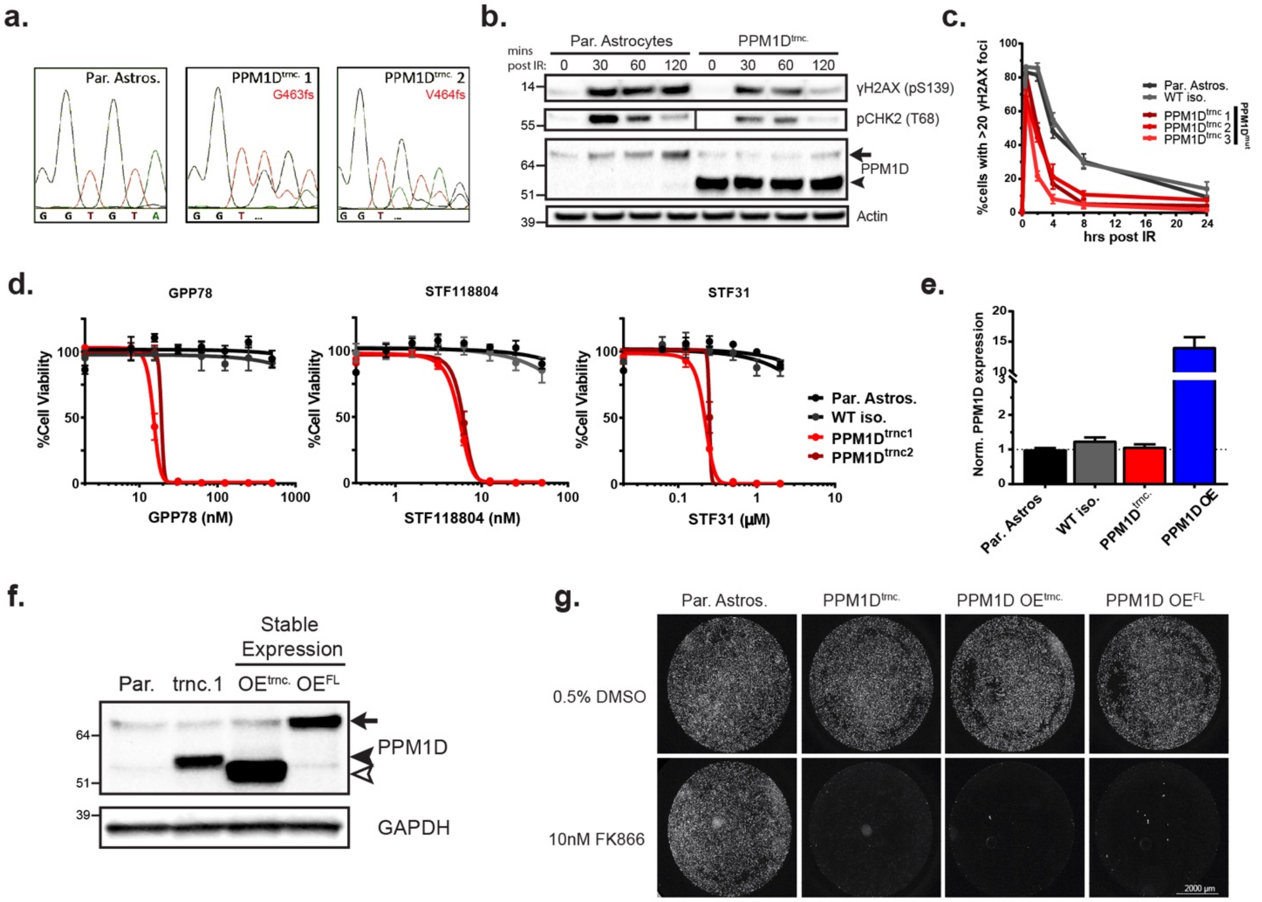

**Supplementary Fig. 1. *PPM1D* mutant astrocytes are sensitive to NAMPT inhibitors.**
(**a**) Sequencing chromatograms within a region of *PPM1D* exon 6 from parental and PPM1D$^{trnc.}$ cell lines. (**b**) Immunoblot of parental and PPM1D$^{trnc.}$ cell lines in response to radiation. Full length (full arrow) and CRISPR-modified (arrowhead) sizes of PPM1D displayed.
(**c**) Quantification of γH2AX foci post radiation (IR) (n=4 independent samples). (**d**) Viability assessments of cell lines after 72hr treatment with three different NAMPT inhibitors (GPP78, STF118804, and STF31) (n =3 independent samples). (**e**) Quantification of *PPM1D* transcript levels in astrocyte cell lines (n =4 independent samples). (**f**) Immunoblot of astrocytes with stable expression of wild type (OE$^{FL}$) or mutant (OE$^{trnc.}$) PPM1D. Full length (full arrow), CRISPR-edited (black arrowhead), and ectopically-expressed mutant protein (white arrowhead) sizes of PPM1D are displayed. (**g**) Representative wells of H33342-stained nuclei from parental and mutant astrocytes, 72hrs post DMSO or FK866 treatment. Error bars represent standard deviation of the mean.

**Supplementary Figure 2.**

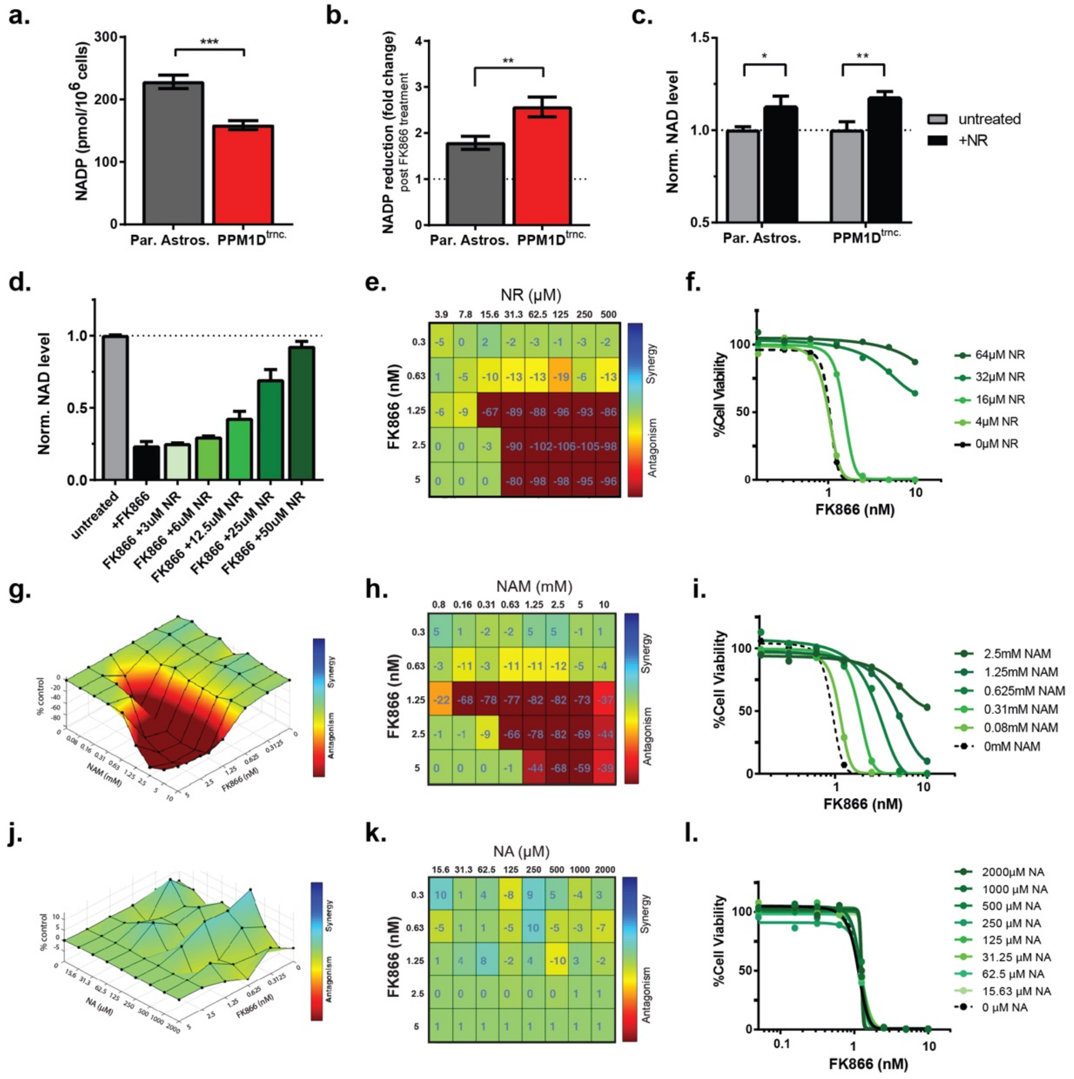


**Supplementary Fig. 2. NAD metabolome depression in PPM1D$^{trnc.}$ astrocytes results in NAMPT inhibitor sensitivity.** (**a**) NADP quantification in parental and PPM1D$^{trnc.}$ astrocytes (n=3 independent samples, *** p<0.001 by Student's T test). (**b**) Relative fold change in NADP levels after treatment with 10nM FK866 for 24hrs (n=3 independent samples, ** p<0.01 by Student's T test). (**c**) NAD quantification after exogenous addition of 50μM nicotinamide riboside (NR) for 24 hrs (n=3 independent samples, * p<0.05, ** p<0.01 by Student's T test). (**d**) Normalized NAD levels in astrocytes after 24hr treatment with 10nM FK866 and indicated doses of NR (n=2 independent samples). (**e**) Bliss model matrix for the antagonistic effects of NR on FK866 treatment in PPM1D$^{trnc.}$ astrocytes. (**f**) Viability assessment of PPM1D$^{trnc.}$ astrocytes after 72hr concurrent FK866 and NR treatment. (**g, j**) Bliss 3D surface plots modelling the antagonistic effects of NAM (g) or NA (j) on FK866 treatment in PPM1D$^{trnc.}$ astrocytes. (**h, k**) Bliss model matrices for the antagonistic effects of NAM (h) or NA (k) on FK866 treatment in PPM1D$^{trnc.}$ (**i, l**) Viability assessment of PPM1D$^{trnc.}$ astrocytes after 72hr concurrent treatment of FK866 with NAM (i) or NA (l). Error bars represent standard deviation of the mean.

**Supplementary Figure 3.**

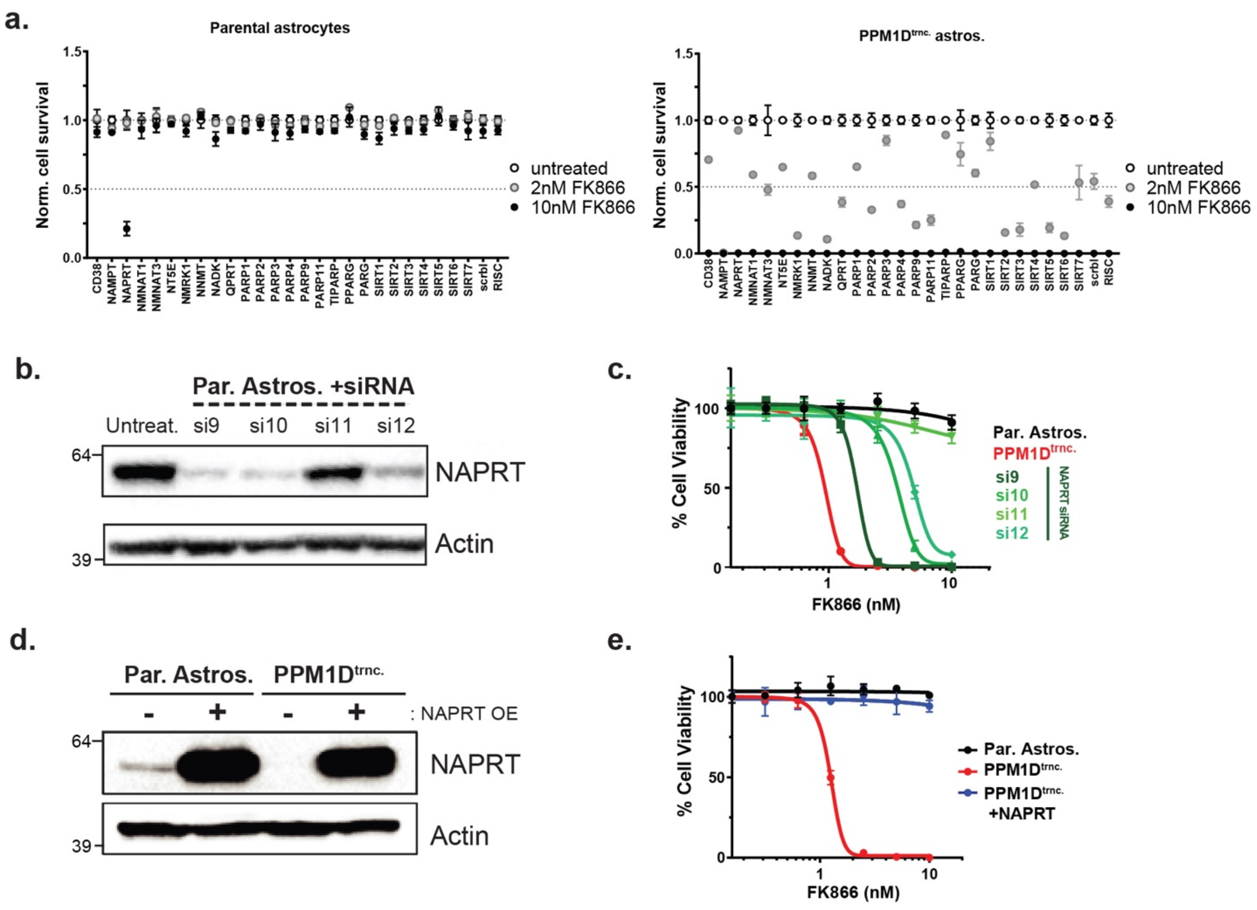


**Supplementary Fig. 3. NAPRT deficiency drives sensitivity of *PPM1D* mutant astrocytes to NAMPT inhibitors.** (**a**) Normalized viability of parental (*left*) and PPM1D$^{trnc.}$ (*right*) astrocytes to FK866 treatment after transfection with a panel of siRNAs targeting NAD biosynthesis-related enzymes (n=2 independent samples). (**b**) Immunoblot of NAPRT protein level after treatment with different NAPRT-targeted siRNAs. (**c**) Viability analysis of cell lines in b., treated with FK866 for 72hrs (n=4 independent samples). (**d**) Immunoblot of parental and PPM1D$^{trnc.}$ astrocytes +/- stable expression of NAPRT. (**e**) Viability assessment Par. Astros., PPM1D$^{trncs.}$, and a NAPRT-expressing PPM1D$^{trnc.}$ (PPM1D$^{trnc.}$+ NAPRT) cell line upon 72hr FK866 treatment (n=4 independent samples). Error bars represent standard deviation of the mean.

**Supplementary Figure 4.**

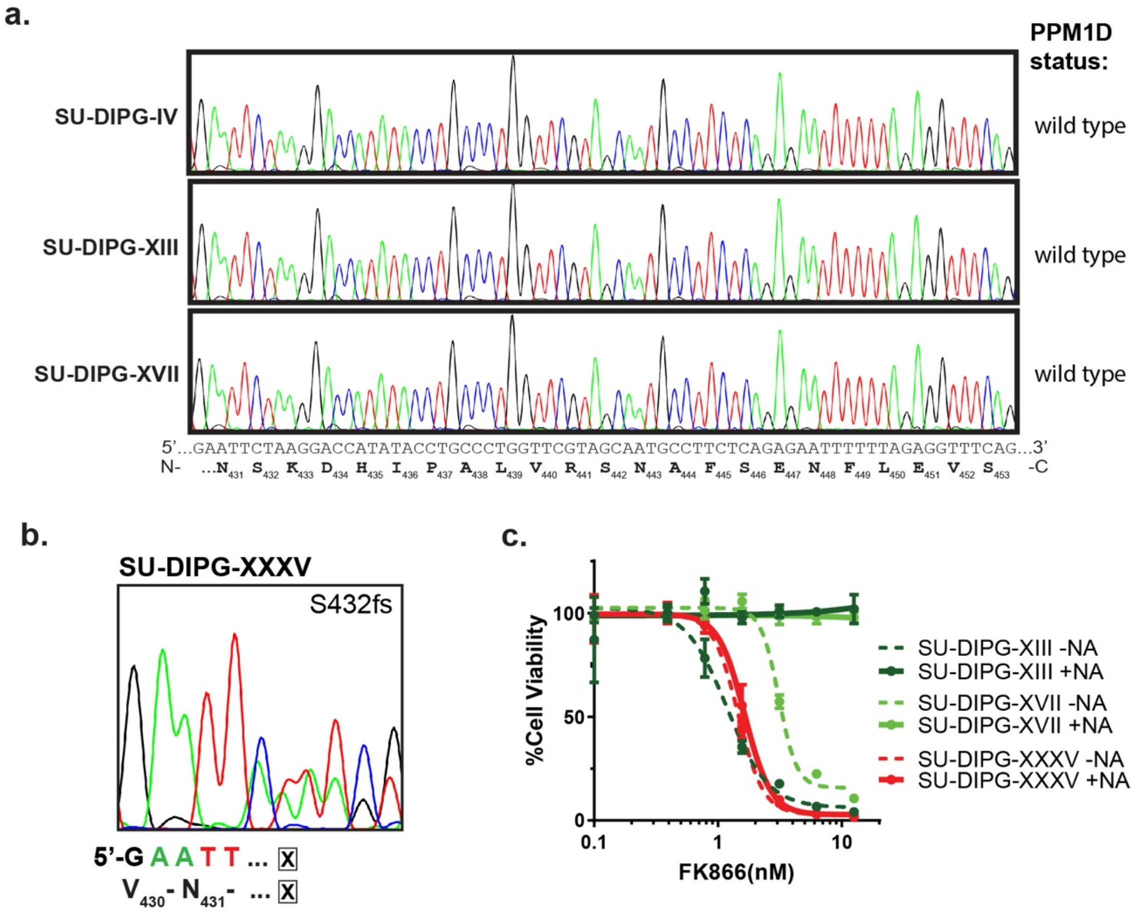


**Supplementary Fig. 4. Patient-derived SU-DIPG-XXXV spheroid cell line possesses a truncating PPM1D mutation and is sensitive to NAMPT inhibitors**. (**a**) Sequencing chromatograms within a region of *PPM1D* exon 6, from SU-DIPG-IV, XIII, and XVII spheroid cell lines. (**b**) Chromatogram of PPM1D-truncating mutation in SU-DIPG-XXXV. (**c**) Viability assessments of SU-DIPG spheroids to FK866 in nicotinic acid (NA) containing (+NA) or NA lacking (-NA) culture media (n=3 independent samples). Error bars represent standard deviation of the mean.

**Supplementary Figure 5.**

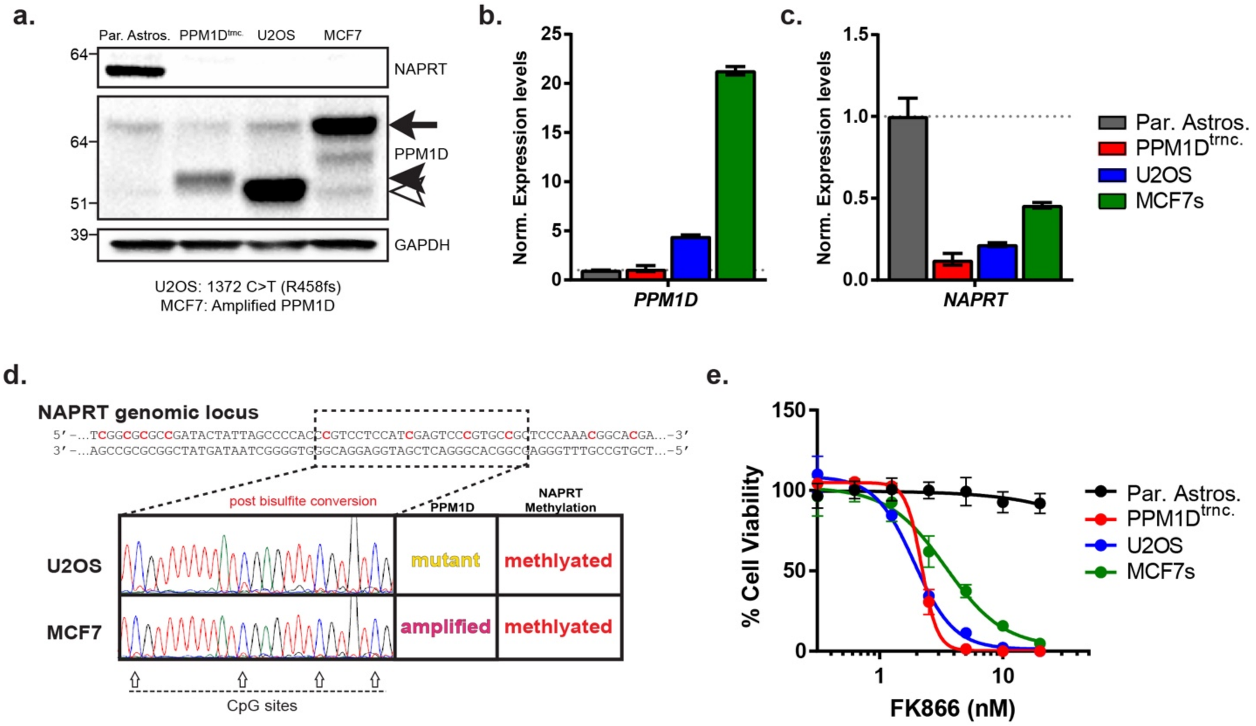


**Supplementary Fig. 5. U2OS and MCF7 cell lines contain *PPM1D* alterations, silence *NAPRT* transcription, and are sensitive to NAMPT inhibitors.** (**a**) Immunoblot of isogenic astrocytes, U2OS, and MCF7 cell lines. (**b, c**) Normalized mRNA expression of *PPM1D* (b) and *NAPRT* (c) in cell panel from a (n=4 independent samples). Error bars represent 95% Confidence Interval about the mean. (**d**) Sequencing chromatograms of the *NAPRT* promoter within U2OS and MCF7 cell lines after bisulfite conversion; arrows indicate potential CpG methylation sites. (**e**) Viability assessment of isogenic astrocytes, U2OS, and MCF7 cell lines after 96hr treatment with FK866 (n=3 independent samples). Error bars represent standard deviation of the mean.

**Supplementary Figure 6.**

**a.**

| DIPG line | PPM1D | Histone 3 mutation | p53 |
|---|---|---|---|
| HSJD-DIPG-007 | P428fs | H3.3 (K27M) | WT |
| HSJD-DIPG-008 | R429fs | H3.3 (K27M) | WT |
| HSJD-DIPG-14b | L484fs | H3.3 (K27M) | WT |
| SU-DIPG-IV | WT | H3.1 (K27M) | WT |
| SU-DIPG-XIII | WT | H3.3 (K27M) | WT |
| SU-DIPG-XVII | WT | H3.3 (K27M) | ND |
| SU-DIPG-XXXV | S432fs | H3.3 (K27M) | WT |

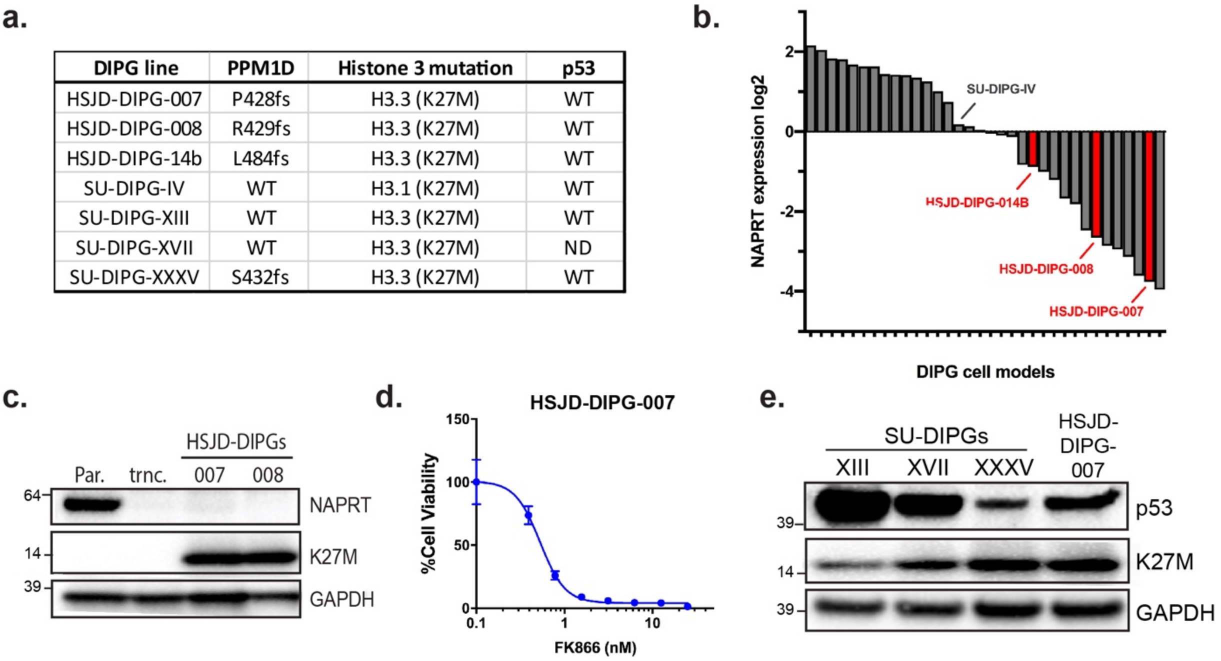


**Supplementary Fig. 6. DIPG model cell lines with *PPM1D* mutations have reduced NAPRT expression and maintain p53 expression.** (**a**) Table depicting mutational status of patient-derived DIPG cell lines in Fig 3e; ND indicates no data available. (**b**) *NAPRT* expression levels of model DIPG cell lines. (**c**) Immunoblot of select astrocyte and DIPG cell lines for NAPRT and H3K27M expression. (**d**) Viability of HSJD-DIPG-007 cell line after 120hr of treatment with FK866 (n=5 independent samples). Error bars represent standard deviation of the mean. (**e**) Immunoblot of DIPG cell line panel for p53 and H3K27M expression.

**Supplementary Figure 7.**

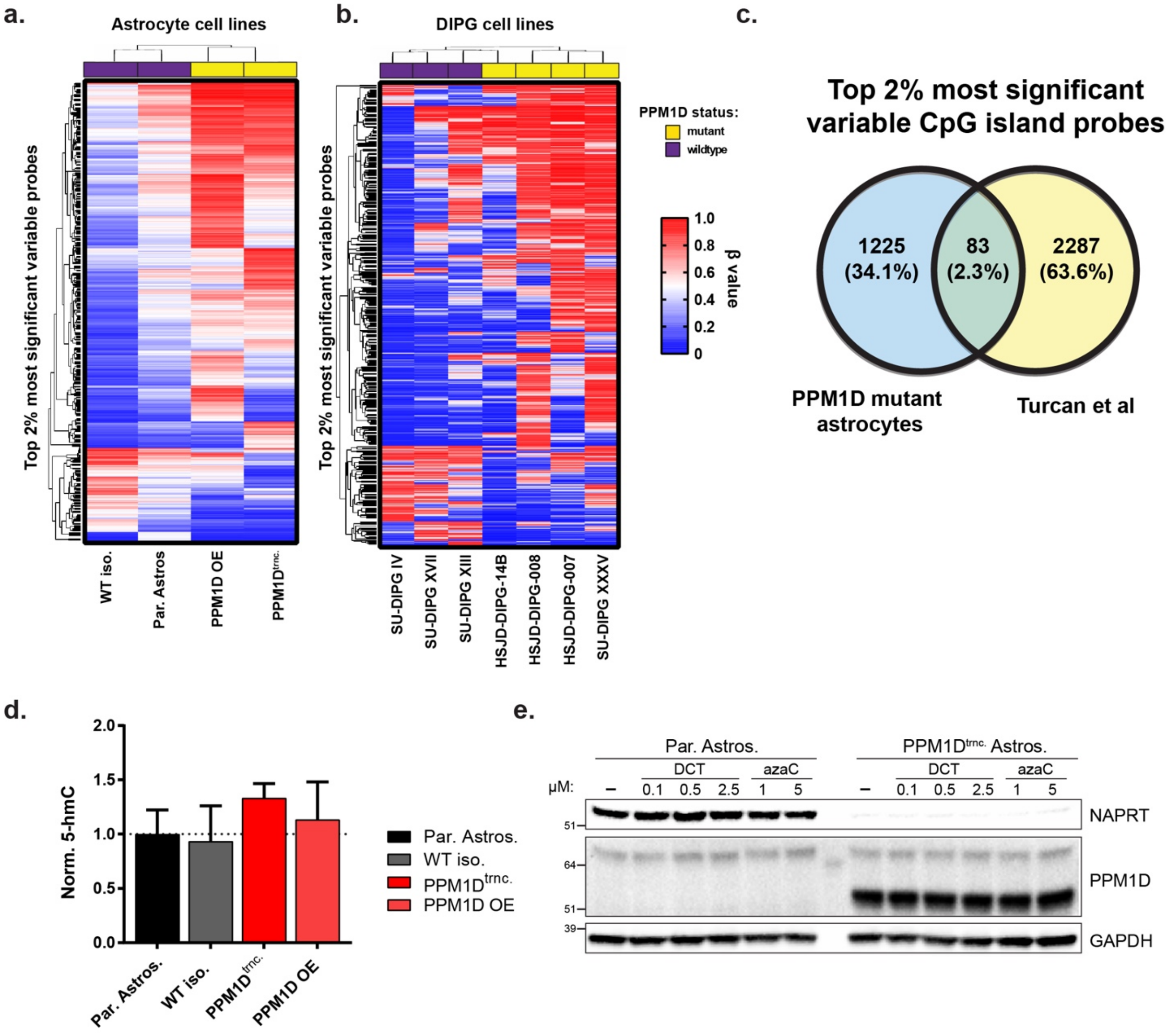


**Supplementary Fig. 7. Mutant PPM1D-induced hypermethylation is distinct from G-CIMP found in *IDH1* mutant astrocytes.** (**a, b**) Hierarchical clustering of the top 2% of significantly variable methylation probes in astrocyte (a) and DIPG (b) cell lines. (**c**) Comparison of top 2% significantly variable CpG island probe sets in *PPM1D* mutant- and *IDH1* mutant astrocytes (*30*). (**d**) Normalized levels of global 5-hydroxymethylcytosine in WT and *PPM1D* mutant astrocytes (n=4 independent samples). Error bars represent 95% Confidence Interval about the mean. (**e**) Immunoblot of parental and PPM1D$^{trnc,}$ astrocytes after treatment with varying doses of decitabine (DCT) or azacytidine (azaC) for 72hrs.

## Supplementary Figure 8.

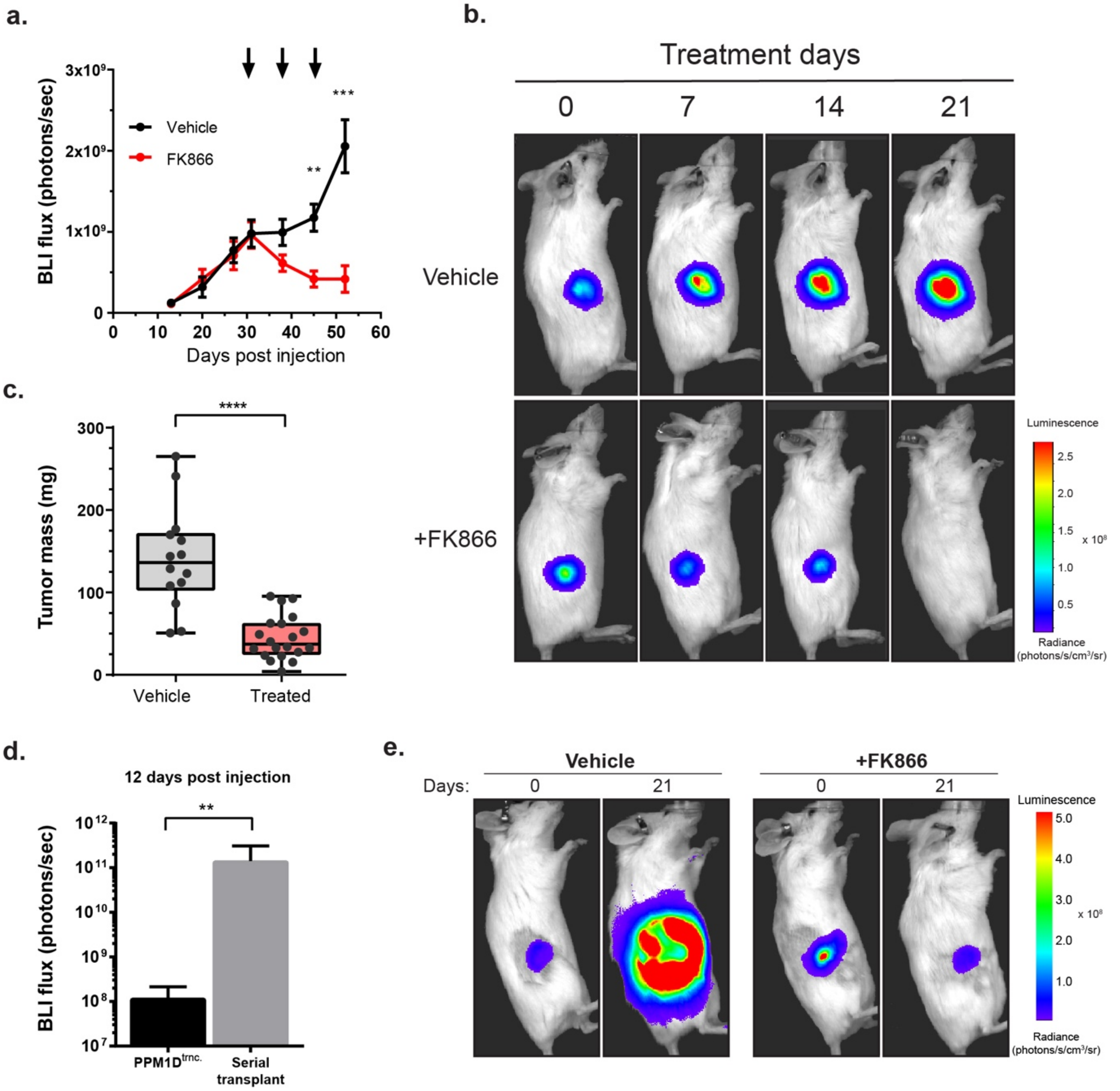


**Supplementary Fig. 8. *In vivo* efficacy of NAMPT inhibitors in *PPM1D* mutant tumors.** (**a**) PPM1D$^{trnc.}$ tumor burden as a measure of bioluminescence imaging (BLI) signal, in NOD *scid* gamma mice treated with vehicle or 20mg/kg FK866 BID for 3 four day cycles as indicated by arrows (n=10 independent animals, error bars represent SE, ** $p<0.01$, *** $p<0.001$ by Mann-Whitney U test). (**b**) Representative BLI images of vehicle and FK866-treated mice over course of treatment. (**c**) Tumor mass measurements, from extracted tumors in a., 2 months post injection (n=14 independent tumors, **** $p<0.0001$ by Student's T test). (**d**) Comparison of BLI signal intensity between PPM1D$^{trnc.}$ cell line xenografts and serially-transplanted *PPM1D* mutant xenografts, 12 days post injection (n=17 independent tumors, ** $p<0.01$ by Student's T test). Error bars represent standard deviation of the mean. (**e**) Representative BLI images of serially-transplanted *PPM1D* mutant xenografts before or after 3 weeks of indicated treatment.

**Supplementary Figure 9.**

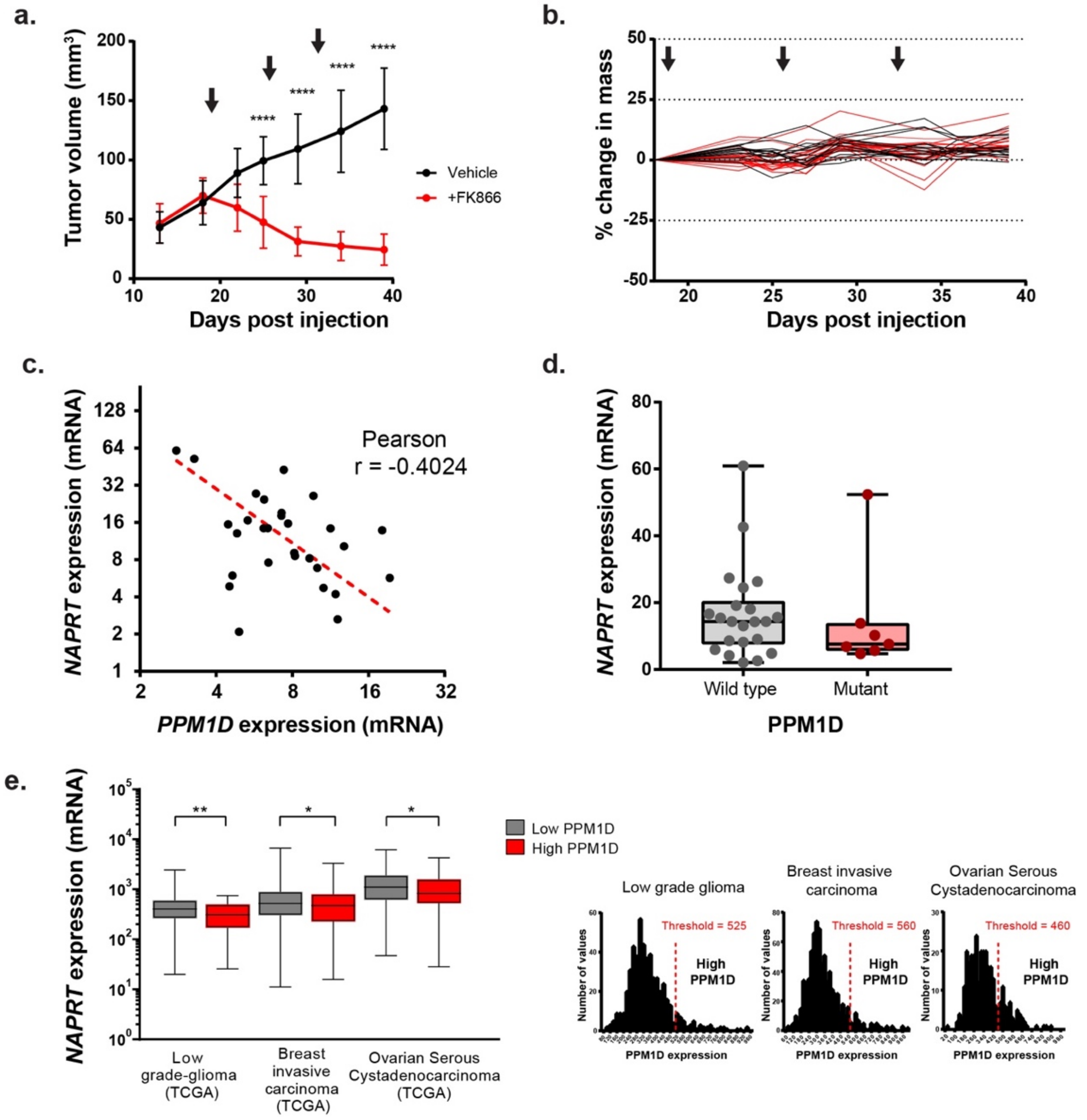


**Supplementary Fig. 9. Applicability of NAMPT inhibitors for the treatment of *PPM1D* mutant, non-glioma tumors.** (**a**) Tumor volume measurements of vehicle or FK866-treated athymic nude mice harboring U2OS cell line xenografts. FK866 treatment consisted of 20mg/kg BID for 3 four day weekly cycles, indicated by arrows (n=15 independent animals, **** $p<0.0001$ by Mann-Whitney U test). Error bars represent standard deviation of the mean. (**b**) Percent change in body mass, measured for each mouse during the duration of treatment described in a. (**c**) *NAPRT* and *PPM1D* expression levels from PNOC003 DIPG cohort (*32*) tumor samples. (**d**) Comparison of *NAPRT* expression levels in wild type and *PPM1D* mutant DIPG tumors from the cohort in c. (**e**) Comparison of *NAPRT* expression levels in *PPM1D* high and low expressing tumors, in cancer subtypes commonly found to have amplification of *PPM1D* (*left*); with histograms of *PPM1D* expression (*right*). * $p<0.05$ ** $p<0.01$ by Student's T test.

## Supplementary Table 1. Synthetic lethal drug screen compounds and $IC_{50}$ ratios.

| Drug Name | Company (catalog) | Target | $IC_{50}\left(\frac{\text{Par. Astros.}}{\text{PPM1D}^{\text{trncs.}}}\right)$ |
|---|---|---|---|
| FK866 | Selleckchem (S2799) | NAMPT | 9746.59 |
| Aphidicolin | Tocris (5736) | Topoisomerase 2 | 1.75 |
| TH287 | Selleckchem (S7631) | MTH1 | 1.52 |
| ETP 45658 | Tocris (4702) | DNApk | 1.51 |
| TMZ | Selleckchem (S1237) | DNA damage | 1.37 |
| SP2509 | Selleckchem (S7680 ) | LSD1 | 1.36 |
| Olaparib | Selleckchem (S1060) | PARP | 1.32 |
| MMS | Sigma (129925) | DNA damage | 1.28 |
| RITA | Selleckchem (S2781) | p53 | 1.27 |
| NU-7441 | Selleckchem (S2638) | DNApk, others | 1.26 |
| KU-55933 | Selleckchem (S1092) | ATM | 1.18 |
| Dexrazoxane | Selleckchem (S5651 ) | Blocks mitosis | 1.17 |
| TCS2312 | Tocris (3038) | CHK1 | 1.13 |
| Lomustine | Selleckchem (S1840) | DNA damage | 1.10 |
| MMC | Selleckchem (S8146) | DNA damage | 1.08 |
| Bendamustine | Selleckchem (S1212) | DNA damage | 1.07 |
| MLM324 | Selleckchem (S7296) | JMJD2 | 1.02 |
| BEZ-235 | Selleckchem (S1009) | PI3K and mTOR | 1.01 |
| ATRN-19 | Atrin Pharm. | ATR | 1.01 |
| Irinotecan | Selleckchem (S2217) | Topoisomerase 1 | 1.01 |
| AZD6482 | Selleckchem (S1462 ) | DNApk, others | 1.00 |
| Etoposide | Selleckchem (S1225) | Topoisomerase 2 | 1.00 |
| GSK2879552 | Selleckchem (S7796) | LSD1 | 1.00 |
| BMN673 | Selleckchem (S7048) | PARP | 0.99 |
| Topotecan | Selleckchem (S1231 ) | Topoisomerase 1 | 0.99 |
| LSD1-C76 | Xcessbio (M66045-2s) | LSD1 | 0.98 |
| PIK 75 | Selleckchem (S1205) | DNApk, others | 0.97 |
| NCS | Sigma (N9162) | DNA damage | 0.94 |
| VE822 | Selleckchem (S7102 ) | ATR | 0.93 |
| MLN4924 | Selleckchem (S7109) | NAE (NHEJ) | 0.92 |
| Cyclophosphamide | Selleckchem (S2057) | DNA damage | 0.90 |
| PD 407824 | Tocris (2694) | CHK1/Wee1 | 0.83 |
| TC-S 7010 | Selleckchem (S1451) | Aurora A | 0.78 |
| AZD7762 | Selleckchem (S1532) | CHK1/2 | 0.77 |
| KU 0060648 | Selleckchem (S8045) | DNApk, others | 0.69 |
| MK-1775 | Selleckchem (S1525) | Wee1 | 0.63 |

**Supplementary Table 2. Oligos and siRNA target sequences.**

| Name | Type | Sequence |
|---|---|---|
| PPM1D guide RNA top | gRNA oligo | ACACCGTTGAGGGTATGACTACACCTG |
| PPM1D guide RNA bottom | gRNA oligo | AAAACAGGTGTAGTCATACCCTCAACG |
| PPM1D gDNA sequencing (forward) | primer | GCATAGATTTGTTGAGTTCTGGG |
| PPM1D gDNA sequencing (reverse) | primer | AGCCCTCTTATATCCTAAGTTTGG |
| PPM1D site-directed mutagenesis | primer | CCAGTCAAGTCACTCGAGGAGGATCCATGA CCAAGGGTGAATTC |
| PPM1D site-directed mutagenesis | primer | GAATTCACCCTTGGTCATGGATCCTCCTCGA GTGACTTGACTGG |
| NAPRT promoter bisulfite sequencing (forward) | primer | CACCTCTGGTGACCAAGACC |
| NAPRT promoter bisulfite sequencing (reverse) | primer | GTGGCCTGGTAGAGGTCAGT |
| NAPRT qPCR (forward) | primer | CGAGAGGAGTTGGGTGACATCC |
| NAPRT qPCR (reverse) | primer | CCTATGGCGCACTCCCTGTG |
| NAPRT siRNA 9 | target sequence | GCAACAACAUUGACGAGGA |
| NAPRT siRNA 10 | target sequence | CUGGAAACAACACGAAUCA |
| NAPRT siRNA 11 | target sequence | GGUAGAGCCCUGACUGGGA |
| NAPRT siRNA 12 | target sequence | GGACAGUGGUGACCUGCUA |
