# Supplementary material for: PPM1D mutations silence NAPRT gene expression and confer NAMPT inhibitor sensitivity in glioma
Source: Nat Commun. 2019 Aug 22;10:3790. doi: 10.1038/s41467-019-11732-6 (PMC6706443; doi:10.1038/s41467-019-11732-6)
Supplement: Supplementary file 2 — Reporting Summary [file 41467_2019_11732_MOESM2_ESM.pdf]

nature research

Corresponding author(s): Ranjit S. Bindra
Michael E. Berens
Charles Brenner

Last updated by author(s): 2019-7-14

Reporting Summary

Nature Research wishes to improve the reproducibility of the work that we publish. This form provides structure for consistency and transparency in reporting. For further information on Nature Research policies, see Authors & Referees and the Editorial Policy Checklist.

Statistics

For all statistical analyses, confirm that the following items are present in the figure legend, table legend, main text, or Methods section.

n/a	Confirmed	
☐	☒	The exact sample size (*n*) for each experimental group/condition, given as a discrete number and unit of measurement
☐	☒	A statement on whether measurements were taken from distinct samples or whether the same sample was measured repeatedly
☐	☒	The statistical test(s) used AND whether they are one- or two-sided<br>*Only common tests should be described solely by name; describe more complex techniques in the Methods section.*
☐	☒	A description of all covariates tested
☐	☒	A description of any assumptions or corrections, such as tests of normality and adjustment for multiple comparisons
☐	☒	A full description of the statistical parameters including central tendency (e.g. means) or other basic estimates (e.g. regression coefficient) AND variation (e.g. standard deviation) or associated estimates of uncertainty (e.g. confidence intervals)
☐	☒	For null hypothesis testing, the test statistic (e.g. *F*, *t*, *r*) with confidence intervals, effect sizes, degrees of freedom and *P* value noted<br>*Give P values as exact values whenever suitable.*
☒	☐	For Bayesian analysis, information on the choice of priors and Markov chain Monte Carlo settings
☐	☒	For hierarchical and complex designs, identification of the appropriate level for tests and full reporting of outcomes
☐	☒	Estimates of effect sizes (e.g. Cohen's *d*, Pearson's *r*), indicating how they were calculated

Our web collection on statistics for biologists contains articles on many of the points above.

Software and code

Policy information about availability of computer code

Data collection	The softwares: CellProfiler (version 3.0.0) , GenomeStudio 2011.1 (version 1.9) , ImageJ (version 1.8.0) and Combenefit (version 2.021) , were used in the collection of data.
Data analysis	Microsoft Excel 2016, GraphPad Prism 7, and R were used in the analysis and graphing of data.

For manuscripts utilizing custom algorithms or software that are central to the research but not yet described in published literature, software must be made available to editors/reviewers. We strongly encourage code deposition in a community repository (e.g. GitHub). See the Nature Research guidelines for submitting code & software for further information.

Data

Policy information about availability of data

All manuscripts must include a data availability statement. This statement should provide the following information, where applicable:
- Accession codes, unique identifiers, or web links for publicly available datasets
- A list of figures that have associated raw data
- A description of any restrictions on data availability

Figures from Infinium Methylation array have associated raw data, and are available at NCBI GEO GSE134165

Field-specific reporting

Please select the one below that is the best fit for your research. If you are not sure, read the appropriate sections before making your selection.

☒ Life sciences ☐ Behavioural & social sciences ☐ Ecological, evolutionary & environmental sciences

Life sciences study design

All studies must disclose on these points even when the disclosure is negative.

Sample size	Sample sizes were determined empirically, from pilot studies, and were chosen to provide sufficient statistical power for the different types of analysis to be performed.
Data exclusions	No in vitro data was excluded, and only two mice were excluded from these studies (one PPM1Dtrnc. xenograft, one U2OS xenograft). These mice were excluded for failure to form measurable tumors after 45 days post flank injection, prior to dosing.
Replication	In vitro findings were repeated at least twice to confirm results. In vivo studies were expanded and replicated from pilot experiments, which served as initial proof of concept models.
Randomization	Animal groups were determined by randomized sorting, post first tumor measurement, but prior to initiation of treatment
Blinding	Animal measurement and analysis were performed by two different individuals, and analysis was blinded from treatment conditions.

Reporting for specific materials, systems and methods

We require information from authors about some types of materials, experimental systems and methods used in many studies. Here, indicate whether each material, system or method listed is relevant to your study. If you are not sure if a list item applies to your research, read the appropriate section before selecting a response.

Materials & experimental systems

n/a	Involved in the study	
☐	☒	Antibodies
☐	☒	Eukaryotic cell lines
☒	☐	Palaeontology
☐	☒	Animals and other organisms
☒	☐	Human research participants
☒	☐	Clinical data

Methods

n/a	Involved in the study	
☒	☐	ChIP-seq
☒	☐	Flow cytometry
☒	☐	MRI-based neuroimaging

Antibodies

Antibodies used	PPM1D (SCBT F-10 sc-376257), GAPDH (Proteintech group HRP-60004), Actin (ThermoFisher MA5-11869), H2AX pS139 (CST 2577), NAPRT (Proteintech group 66159-1), NAMPT (CST D7V5J 86634), pCHK2 T68 (CST 2197), H3K4me3 (CST 9751), H3K27ac (Abcam ab4729), H3K4me1 (Abcam ab8895), H3K27me3 (CST 9733), H3K27M (CST 74829), and p53 (CST 9282)
Validation	PPM1D: transfected control lysates (SCBT)<br>GAPDH: control cell lines (Proteintech group)<br>Actin: control cell line and tissue staining (ThermoFisher)<br>H2AX pS139: control lysates UV treatment controls (CST)<br>NAPRT: control cell line and tissue staining (Proteintech group)<br>NAMPT: control cellular lysates (CST)<br>pCHK2 T68: control lysates and tissue, UV treatment controls (CST)<br>H3K4me3: control lysates, competition assays, control cell lines, ChIP testing (CST)<br>H3K27ac: control cell lines and lysates, ChIP titration (abcam)<br>H3K4me1: control cell lines and lysates, ChIP titration, competition assays (abcam)<br>H3K27me3: control cell line and tissue staining, ChIP testing (CST)<br>H3K27M: control cellular lysates (CST)<br>p53: control cell lines and cellular lysates (CST)

Eukaryotic cell lines

Policy information about cell lines

Cell line source(s)	Immortalized human astrocytes were obtained from the lab of Dr. Timothy Chan. SU-DIPG spheroids lines were obtain from the lab of Dr. Michelle Monje. U2OS cell lines were acquired from ATCC. MCF7 cells were a gift from the lab of Dr. Peter Glazer. HSJD-DIPG-007 and -008 neurosphere lines were acquired from the labs of Drs. Chris Jones and Angel M. Carcaboso.
Authentication	Immortalized human astrocytes were authenticated via STR (GRCF-John's Hopkins). Other cell lines in the study were not authenticated upon acquisition.

Mycoplasma contamination	Cell lines tested negative for mycoplasma contamination.
Commonly misidentified lines (See ICLAC register)	N/A

Animals and other organisms

Policy information about studies involving animals; ARRIVE guidelines recommended for reporting animal research

Laboratory animals	Female, NSG (NOD.Cg-Prkdcscid Il2rgtm1Wjl/SzJ) mice were used for astrocyte xenograft studies, ages 4-6 weeks. Female athymic nude mice were used in U2OS xenograft studies, ages 4-6 weeks.
Wild animals	Studies did not involved wild animals
Field-collected samples	Studies did not involves samples collected from the field.
Ethics oversight	All animal use was in accordance with the guidelines of the Animal Care and Use Committee (IACUC) of Yale University and conformed to the recommendations in the Guide for the Care and Use of Laboratory Animals (Institute of Laboratory Animal Resources, National Research Council, National Academy of Sciences, 1996).

Note that full information on the approval of the study protocol must also be provided in the manuscript.
